# A plastome primer set for comprehensive quantitative real time RT-PCR analysis of *Zea mays*: a starter primer set for other *Poaceae *species

**DOI:** 10.1186/1746-4811-4-14

**Published:** 2008-06-02

**Authors:** Richard M Sharpe, Sade N Dunn, A Bruce Cahoon

**Affiliations:** 1 Department of Biology, Middle Tennessee State University, Murfreesboro, TN, USA

## Abstract

**Background:**

Quantitative Real Time RT-PCR (q2(RT)PCR) is a maturing technique which gives researchers the ability to quantify and compare very small amounts of nucleic acids. Primer design and optimization is an essential yet time consuming aspect of using q2(RT)PCR. In this paper we describe the design and empirical optimization of primers to amplify and quantify plastid RNAs from *Zea mays *that are robust enough to use with other closely related species.

**Results:**

Primers were designed and successfully optimized for 57 of the 104 reported genes in the maize plastome plus two nuclear genes. All 59 primer pairs produced single amplicons after end-point reverse transcriptase polymerase chain reactions (RT-PCR) as visualized on agarose gels and subsequently verified by q2(RT)PCR. Primer pairs were divided into several categories based on the optimization requirements or the uniqueness of the target gene. An *in silico *test suggested the majority of the primer sets should work with other members of the Poaceae family. An *in vitro *test of the primer set on two unsequenced species (*Panicum virgatum *and *Miscanthus sinensis*) supported this assumption by successfully producing single amplicons for each primer pair.

**Conclusion:**

Due to the highly conserved chloroplast genome in plant families it is possible to utilize primer pairs designed against one genomic sequence to detect the presence and abundance of plastid genes or transcripts from genomes that have yet to be sequenced. Analysis of steady state transcription of vital system genes is a necessary requirement to comprehensively elucidate gene expression in any organism. The primer pairs reported in this paper were designed for q2(RT)PCR of maize chloroplast genes but should be useful for other members of the Poaceae family. Both *in silico *and *in vitro *data are presented to support this assumption.

## Background

Chloroplasts are semi-autonomous organelles believed to have developed from free-living photosynthetic bacteria [1,2] They are members of a diverse and flexible family of organelles called plastids that are responsible for photosynthesis plus other essential biosynthetic pathways and cellular functions. Plastids have maintained a small remnant genome with a species-specific number of genes mostly involved in photosynthesis and gene expression. The full function and development of all the plastid types, however, requires thousands of nuclear encoded gene products.

Maize is an agriculturally important monocot grass that has served as a genetic model system for decades [3], is the focus of a major genome project [4,5], and is especially valuable for the study of chloroplast biology [6]. Other members of the grass family (*Poaceae*) also offer unique opportunities to study differential plastid gene expression. The presence of closely related species with either C3 or C4 photosynthetic capabilities enables comparison of dimorphic C4 chloroplast development to monomorphic C3 species. In addition, grasses such as switchgrass (*Panicum virgatum*) and miscanthus (*Miscanthus sinensis*) are the subjects of an increased focus on cellulosic ethanol production [7].

To date, most *Poaceae *chloroplast gene expression studies have concentrated on proteomic or physiologic assays or focused on post-transcriptional modification and regulation of transcripts, [8-10]. While these approaches elucidate end point development of transcribed genes, they rarely illuminate the transcription activity of a particular gene. q2(RT)PCR is a maturing tool sensitive enough to detect the existence of small amounts of nucleic acid [11]. This allows for in-depth, comprehensive investigations into transcript abundance and offers a useful tool to help elucidate the relationship between transcription, translation, and expression.

q2(RT)PCR is dependent upon the amplification and quantification of a single amplicon that makes primer design and amplification conditions key to the success of an experiment. Substantial time and resources may be spent in the design, testing, and subsequent reworking of primers for optimal efficiency [12].

In this paper we describe the development and empirical optimization of primer pairs to amplify each rRNA and mRNA from maize plastids. Primer sequences, optimal annealing temperatures, and extension times are reported. In addition, each primer set was tested, *in silico*, against published plastome sequences and *in vitro *against switchgrass and miscanthus transcripts. Using the conditions optimized for maize, all primer pairs successfully produced a single amplicon for these two grass species.

## Results and Discussion

**Figure 1:**
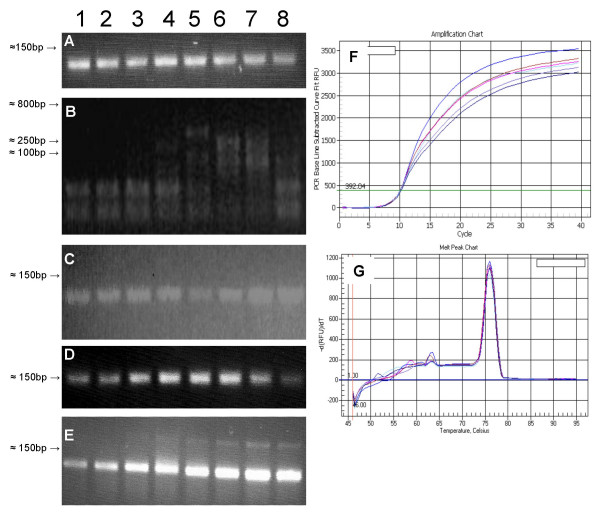
Primer optimization and visual verification of a single amplicon for maize chloroplast genes utilizing endpoint RT-PCR. Lanes 1 through 8 denote an annealing temperature gradient, **Panel A **shows amplification of *psbB *mRNA and represents a typical primer optimization run for a Category 1 primer set. In addition to temperature, *psbB*'s optimization elongation time was 2 minutes. Reducing the elongation time to 30 seconds yielded equivalent results. **Panels B **and **C **show results for *psaB *primer pairs. **Panel B **shows multiple products due to a 2 minute extension time and 5' and 3' primers with a 4°C difference in annealing temperatures. **Panel C **shows end-point PCR results with a 30 second extension time and the 5' and 3' primers re-designed to have melting temperatures with less than 1°C difference. **Panel D **represents the optimized annealing temperatures for a single amplicon of *rpl14 *indicative of category 2a. **Panel E **represents the optimized annealing temperatures for an *atpI *amplicon that is indicative of category 2b. Items **F **and **G **illustrate q2(RT)PCR amplification (**F**) and melt curve (**G**) of a category 1 primer set for *matK *optimized at different annealing temperatures. In **Panel F **the x-axis denotes cycle number, the y-axis denotes the base-line subtracted recorded fluorescence units and demonstrates that overall efficiency of the reactions deviate slightly but the crossover thresholds remain consistent. In **Panel G **the x-axis denotes temperature, the y-axis denotes the change in recorded fluorescence units, and confirms all annealing temperatures produce a single amplicon with a melting temperature of 76.00°C. **Panel A **annealing temperatures 1 = 59.0°, 2 = 58.2°,3 = 57.0°, 4 = 55.1°, 5 = 52.7°, 6 = 50.9°, 7 = 49.7°, 8 = 49.0°. **Panel B **1 = 63.0°, 2 = 62.0°, 3 = 60.1°, 4 = 57.2°, 5 = 53.2°, 6 = 50.4°, 7 = 48.4°, 8 = 47.2°. **Panel C **1 = 60.0°, 2 = 59.3°, 3 = 58.2°, 4 = 56.4°, 5 = 54.3°, 6 = 52.7°, 7 = 51.6°, 8 = 51.0°. **Panel D **1 = 62.0°, 2 = 60.9°, 3 = 59.0°, 4 = 56.1°, 5 = 52.5°, 6 = 49.9°, 7 = 48.0°, 8 = 47.0°. **Panel E **1 = 64.0°, 2 = 63.1°, 3 = 61.6°, 4 = 59.3°, 5 = 56.4°, 6 = 54.3°, 7 = 52.8°, 8 = 52.0°.

One of the challenging and time consuming aspects of q2(RT)-PCR is the design and optimization of primer pairs which yield single amplicons. The aim of this study was to design and optimize a comprehensive set of plastid specific primers for q2(RT)PCR specific enough to yield robust steady state transcript data from maize yet flexible enough to recognize transcripts from multiple members of the family *Poaceae*. To accomplish this, primers were designed against highly homologous protein coding regions from the maize plastome, as well as rRNA genes. Primers were designed to produce amplicons 75–150 bp in length, have similar annealing temperatures, and were carefully evaluated for favorable melting temperatures to insure a lack of intra-molecular folding. Each primer set was qualitatively examined and optimized with endpoint RT-PCR as seen in Figure 1(A–E). These optimal conditions were then utilized in q2(RT)-PCR for each primer pair to verify the amplification of single amplicons via melt curves and their ability to produce quantitative data as seen in figure 1(F) and 1(G). The complete set of primer pairs yielding single amplicons and their optimized conditions is listed in Additional File 1.

**Figure 2:**
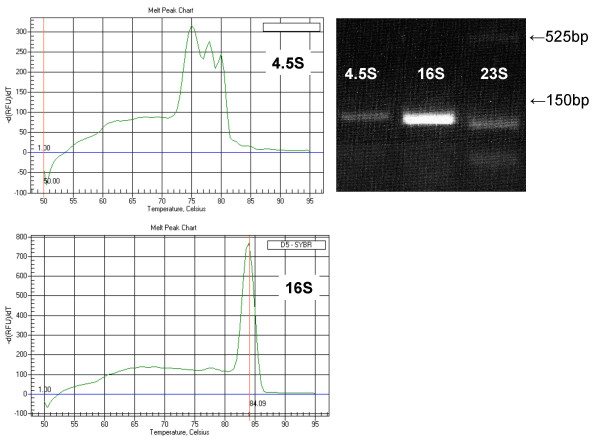
Sensitivity difference between endpoint RT-PCR and q2(RT)PCR for genes found in multiple organelles. Endpoint reveals one product for both *4.5S *utilizing suboptimal primers and *16S *(optimal primers) whereas the q2(RT)PCR melt curve for *4.5S *clearly shows three peaks denoting three products. q2(RT)PCR melt curve for *16S *verified only one product. This primer set for *23S *was not analyzed by q2(RT)PCR due to the presence of multiple endpoint products. A redesign of the *23S *3' primer was accomplished and is reported in Additional File 1.

Throughout the design and optimization process, we found it useful to categorize the primers based on their ease of use and optimal amplification conditions. For example, primer pairs that produced a single amplicon over a relatively large range of annealing temperatures and elongation times as shown in figure 1A were designated category 1 primers. Category 2 primer pairs produced multiple products at non-optimal annealing temperatures or elongation times and are shown in figure 1B,1C,1D, and 1E; category 3 primer pairs produced multiple amplicons due to the presence of multiple organellar genomes or primers having a high affinity to unknown additional nucleotide sequences. Category 1 primer pairs consistently were the most forgiving of strict protocol adherence. Elongation and annealing temperatures could be adjusted with no visible endpoint differences. Category 2 primer pairs required stricter elongation times and annealing temperatures for optimal single product results. Subcategory 2a includes primer pairs where an elongation time greater than 30 seconds resulted in multiple erroneous amplicons. Subcategory 2b includes primer pairs requiring specific annealing temperatures either to generate the most product as seen in Figure 1D or to produce only one product as shown in Figure 1E. One lesson learned is that some primer pairs initially in the 2b subcategory could be moved to category one if the primers were re-designed such that their individual melting temperatures were more similar. Figure 1B illustrates inconsistent and nonspecific product bands from the first pair of primers designed for *psaB *that had individual primer melting point temperatures that differed by 4°C. The subsequent set of primers designed for *psaB *have more similar melting temperatures and optimized end point results can be seen in Figure 1C. The third category of primer pairs consist of gene sequences where a highly similar homolog exists either in the nucleus or mitochondria (*4.5S*, *16S*, *atpE*, *rpl2*, and *23S*) and\or genes where multiple attempts to design primer pairs produced unacceptable products (*rps12 *and *rps7*). Two category 3 examples, *4.5S *and *16S*, are shown in Figure 2. For *4.5S*, an end-point reaction utilizing suboptimal primers appeared to produce a single band but a melt peak curve clearly showed three very similar but distinct products. A subsequent redesign of the 3' primer for 4.5S and 23S resulted in single amplicons verified by their respective melt curve signatures and are reported as the 3' primers in Additional File 1. *16S*, on the other hand, should also produce multiple bands according to screens of the maize genomes but neither end-point nor q2(RT)PCR can distinguish separate products. We could not successfully design some category 3 primer pairs. They either repeatedly produced multiple amplicons or could not be verified as amplified from the chloroplast genome. These primer pairs have been excluded from Additional File 1 or do not have annealing temperatures associated with them.

Some plastid genes with intra-cellular homologs could be specifically amplified when primers were designed to take advantage of small non-similar regions within the genes. This was achieved by comparing the homology between the genes and designing primers to recognize unique regions within chloroplast genes. Primers were then compared to other competing homologs, if the primer homology was less than 50% and there were no 3' matching nucleotides, then the design process was completed and the primers synthesized. The similarity estimation was based on the BLAST [13] percent identity score and not the E value which can be inflated when sequence sizes are small. One exception to this design strategy was the *atpA *gene primers which have >50% homology and should anneal to mitochondrial homologous regions. The possible mitochondrial amplicon however was predicted to be 125,630 base pairs long. An experimental end-point check of the *atpA *primers amplified the desired 77 base pair chloroplast amplicon and the single product was verified by a q2(RT)PCR melt curve. Multi-genomic genes exhibiting high homology but whose primer annealing sites and subsequent amplicons are plastid specific when analyzed in this manner are reported in Additional File 1 as category 1 or category 2 genes. Multi-genomic genes with homologies below 50% and whose primer pairs were determined to amplify only chloroplast genomic material are also reported in Additional File 1 as category 1 or category 2 genes.

**Figure 3:**
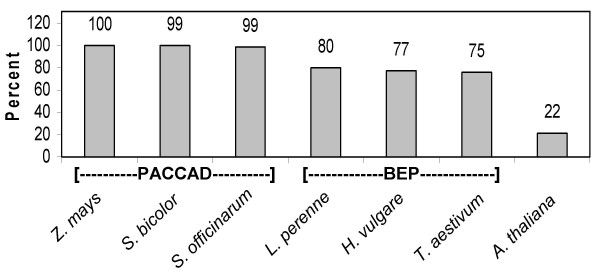
*In silico *comparisons of *Zea mays *plastome primers to other species. Each primer pair was stand alone BLASTed against each plastid genome. A primer was considered a match if no more than two nucleotides were a mismatch and no mismatch could occur within the first four nucleotides of the 3' end. The height of each column represents the percentage of the primers predicted to anneal to the intended target gene or transcript.

The homology between plastomes from closely related plant species is well documented [14-16]. This homology has been previously utilized to design PCR primer pairs that would produce amplicons across genera [17,18]. Based on this, we hypothesized that our primer pairs should amplify similar regions of the same genes from other members of the grass family (*Poaceae*). To test this, an *in silico *comparison of the primers to grasses with completed plastomes was conducted. The possibility of a successful reaction was judged based on overall percent homology and the presence of mismatched bases at the 3' end of the primers. This comparison suggested that primer homology and subsequent amplification success would closely follow established taxonomic designations (Figure 3 and Additional File 2). Members of the PACCAD clade (*S. officinarum *and *S. bicolor*), to which maize belongs, have the greatest possibility of successful amplifications for all primer pairs while there is a noticeable drop in predicted success among members of the BEP clade (*H. vulgare*, *T. aestivum*, *L. perrene*). Arabidopsis was included as an outgroup, with only 22% of the primers predicted to successfully anneal to the intended target gene.

**Figure 4:**
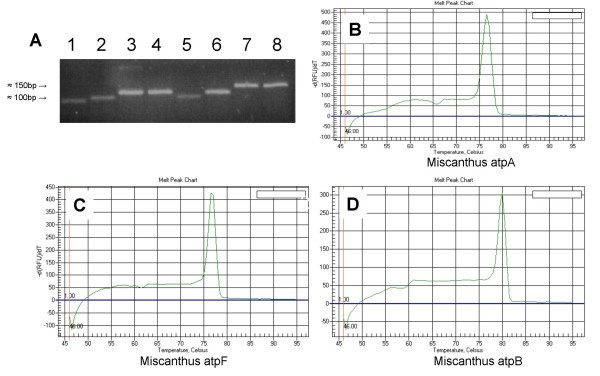
**Panel A**. Endpoint RT-PCR verification of single amplicons for selected Miscanthus genes. 1 – *atpA*, 2 – *atpB*, 3 – *atpE*, 4 – *atpF*, 5 – *atpH*, 6 – *atpI*, 7 – *atpJ*, 8 – *atpK*. **Panel B**. iQ5 q2(RT)PCR verification of single amplicon melt curve analysis for *atpA*. **Panel C**. *atpF ***Panel D**. *atpB*.

**Figure 5:**
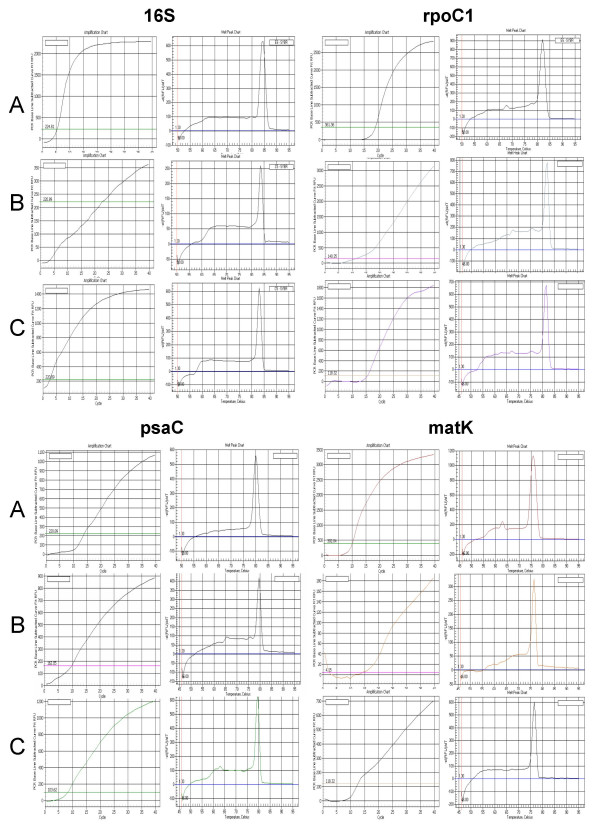
Amplification and melt curve graphs of LSC (*rpoC1 *and *matK*), SSC (*psaC*), and IR (*16S*) representative genes from all three *Poaceae *species. **A**. Maize **B**. Switchgrass **C**. Miscanthus. The left graph of each pair is the PCR amplification curve. The x-axis denotes the PCR cycle number and the y-axis the base-line subtracted recorded fluorescence units. The right chart illustrates the melt curve for each amplicon. The x-axis depicts the temperature in Celsius and the y-axis depicts the change in recorded fluorescence units over time.

The *in silico *results were tested *in vitro *with two species in the PACCAD clade with unsequenced (or unpublished) plastomes – switchgrass (*Panicum virgatum*) and miscanthus (*Miscanthus. sinensis*). As predicted, we found that the primers and their empirically determined optimal conditions worked well across these genera and produced reliable quantitative data [Figure 4]. Representative data from the chloroplast Large Single Copy area (*rpoC1 *and *matK*), Small Single Copy area (*psaC*), and the Inverted Repeat (*16S*) areas are illustrated in Figure 5. As shown, these primer sets and amplification conditions eliminated the need for additional primer design for these species.

As seen in Figure 5, there are obvious differences in expression amounts and amplification slopes between maize (A), switchgrass (B) and miscanthus (C). Although equal amounts of total RNA were added for each round there was no attempt to normalize the data to account for any differences that may exist between species. It also must be stressed that the primer pairs have been optimized for the maize plastome and although all were checked and verified to produce single amplicons for both switchgrass and miscanthus, the reported annealing temperatures were not optimized to ensure the greatest signal was detected for these two species. Individual reagents, specific types of equipment and reaction volumes may require some user-specific modifications for optimal performance for species specific work.

## Conclusion

Primers were designed and empirically optimized to quantify plastid transcripts from *Zea mays*. Due to the highly conserved nature of plastomes within the family *Poaceae*, this set of primers works equally as well for the majority of targeted genes of other species in the Poaceae family, particularly members of the PACCAD clade. This primer set and the conditions published in this report should reduce the time and cost associated with primer design and testing for future plastid q2(RT)PCR experiments.

## Methods

### q2(RT)PCR Primer Design and validation

**Figure 6:**
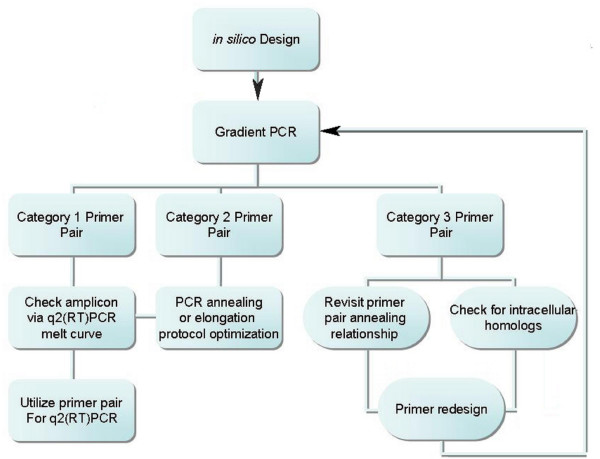
Validation procedural flowchart of q2(RT)PCR primer design.

q2(RT)PCR primer design and validation procedural flow can be seen in Figure 6. Primer pairs were designed from the NCBI published *Zea mays *complete chloroplast genome sequence accession numbers [GenBank: X86563 and GenBank: NC_001666], NAPD-Malic enzyme accession number [GenBank: J05130], and NAPD-Malate dehydrogenase accession number [GenBank: AY105634] [19]. Gene sequences were obtained from each coding sequence (CDS) listing, rRNA listing, or accession number sequence. The coding sequence was then analyzed using the BLAST [13] program for homology to mitochondrial and known nuclear maize genomes. Primers were designed with an attempt to utilize dissimilar sequences between the genomes using the online software primer3 [20]. Suggested primers were checked for inhibitory secondary structures using mFold [21] and edited until the recommended primer produced a positive delta G at 45°C. This ensured selected primer pairs would possess a null or positive delta G energy signature at reverse transcriptase annealing temperatures and amplify chloroplast specific gene sequences. Primer pairs were then optimized to produce a single amplicon of predicted length by end-point temperature gradient PCR with maize whole leaf tissue RNA template and visualized on a 3% TAE agarose gel run alongside a 2-Log ladder from New England Biolabs (Ipswitch, MA). Optimization of primer pairs consisted of running eight temperature gradient dependent reactions and visualizing them on a 3% agarose gel. The gradient spanned a 10°C range, ± 5° from the average melting temperatures (Tm) of the primer pairs as reported by the supply company. Reaction elongation times were set at two minutes in the initial primer set testing phase. Several of the initial primer pairs produced amplicons of greater length than the predicted length. Elongation times for these and subsequent primer pairs were reduced to 30 seconds to better match calculated elongation time [22] for amplicons of the desired size. Amplicons from switchgrass and miscanthus were then verified as the only product via end point RT-PCR visualized on 3% TAE agarose gel and then subsequently verified by the q2(RT)PCR melt curve for a single amplicon with BioRad's IQ™5 Optical System software, Hercules, CA,. All primers were obtained from Integrated DNA Technologies, Coralville, IA.

### RNA material preparation

Maize (inbred line T42) stage 2 leaves were dissected into basal, mid, and tip sectional tissue as described in Cahoon, et al [23] and mixed into a single sample. Switchgrass stem and leaf and miscanthus leaves were harvested from mature demonstration specimens maintained by the MTSU greenhouse. Tissue samples were cut into approximately 2 mm by 2 mm sections, placed in liquid nitrogen, and ground with ceramic mortars and pestles. RNA was extracted with Trizol^® ^according to the manufacturer's suggested protocol, Invitrogen (Carlsbad, CA). Each extraction was checked for a DNA/RNA concentration utilizing a Spectronic^® ^Genesys™ spectrophotometer (Lincoln, NE). Ambion's Turbo DNase-*free*™ or DNase-*free*™, (Applied Biosystems, Foster City, CA) was used according to manufacturers suggested protocol to remove contaminating DNA. DNase treatments were repeated until each RNA extraction was visually confirmed to be DNA free by end point PCR reactions.

### Gene Expression Verification

Each gene primer pair was verified utilizing BioRad's (Hercules, CA) iCycler and IQ™5 Multi-color Real-Time PCR Detection System. 25 μl reactions were prepared with BioRad iScript™ One-Step RT-PCR kit with SYBER^®^Green utilizing approximately 200 ng/ul cleaned RNA per reaction for each of the three tissue samples. Amplicon presence and purity was confirmed by analysis of the IQ™5 Optical System's melt curve, melt curve peak, and PCR amplification software.

## Abbreviations

RT-PCR: reverse transcriptase PCR; q2(RT)PCR: quantitative real time reverse transcriptase PCR

## Competing interests

The authors declare that they have no competing interests.

## Authors' contributions

RMS designed the primers, performed the q2(RT)PCR reactions, interpreted reaction results and prepared the manuscript, SND processed and performed the q2(RT)PCR for the majority of the switchgrass samples, ABC was involved in the conception and design of the project, revised and edited the manuscript, and is the PI of the laboratory.

## Supplementary Material

Additional File 1 q2(RT)PCR primer pairs with their corresponding genes, category, and optimal elongation time, and annealing temperatures. All primers were initially designed to produce amplicons between 75 and 150 base pairs from specific maize RNAs. Primer pair "category" is based on the specificity of the reaction conditions required to produce a single amplicon. Category 1 pairs produce a single product within a range of conditions, category 2 pairs require more stringent annealing or elongation times, and category 3 are genes with intercellular homologs (see text for full descriptions). Click here for file

Additional File 2 *in silico *comparison of maize primers to other plastome sequences. Primer sequences were compared to representative plastomes from the BEP and PACCAD clades, as well as Arabidopsis. Success was gauged by overall percent homology and the presence of mismatched bases at the 3' end of the primers. These data are summarized in Figure 3. Click here for file
