# Supplementary material for: A plastome primer set for comprehensive quantitative real time RT-PCR analysis of Zea mays: a starter primer set for other Poaceae species
Source: Plant Methods. 2008 Jun 2;4:14. doi: 10.1186/1746-4811-4-14 (PMC2453112; doi:10.1186/1746-4811-4-14)
Supplement: Additional File 1 — q2(RT)PCR primer pairs with their corresponding genes, category, and optimal elongation time, and annealing temperatures. All primers were initially designed to produce amplicons between 75 and 150 base pairs from specific maize RNAs. Primer pair "category" is based on the specificity of the reaction conditions required to produce a single amplicon. Category 1 pairs produce a single product within a range of conditions, category 2 pairs require more stringent annealing or elongation times, and category 3 are genes with intercellular homologs (see text for full descriptions). [file 1746-4811-4-14-S1.doc]

Table 1. Primer pairs with their corresponding genes, category, elongation time, and annealing temperatures.

| Gene Name | Primer sequence (forward and reverse) | Category | Optimal elongation time | Optimal annealing temperature (° C)  All annealing times are 30 sec |
| --- | --- | --- | --- | --- |
| atpA ^†^ [CDS](http://www.ncbi.nlm.nih.gov/entrez/viewer.fcgi?val=11990232&from=36573&to=38096&view=gbwithparts) | 5’ acccatcgcccattaataca  3’ aatttgcagaagggacgaga | 1 | 2 min to 30 sec | 56.4 |
| atpB^†^  [CDS](http://www.ncbi.nlm.nih.gov/entrez/viewer.fcgi?val=11990232&from=54618&to=56114&view=gbwithparts) | 5’ gggttgatgagaggaatgga  3’ cgttaaaaattcgtccgaga | 2a | 30 sec | 59.3 |
| atpE^†^ [CDS](http://www.ncbi.nlm.nih.gov/entrez/viewer.fcgi?val=11990232&from=54208&to=54621&view=gbwithparts) | 5’ cggttctgtggagtggtttt  3’ gcctgttgagcttcttctgg | 3 |  |  |
| atpF^†^ [CDS](http://www.ncbi.nlm.nih.gov/entrez/viewer.fcgi?val=11990232&itemID=18&view=gbwithparts) | 5’ctgggagtttcgggcttaat  3’ aactcgcacacactcccttt | 2a | 30 sec | 56.4 |
| atpH^†^ [CDS](http://www.ncbi.nlm.nih.gov/entrez/viewer.fcgi?val=11990232&from=34381&to=34626&view=gbwithparts) | 5’cagaagcagaaggtaaaataagagg  3’ tgccacaaccagtccataaa | 2a | 30 sec | 54.3 |
| atpI^†^  [CDS](http://www.ncbi.nlm.nih.gov/entrez/viewer.fcgi?val=11990232&from=32820&to=33563&view=gbwithparts) | 5’ccaaccccaatccttttacc  3’ cgactaattcatccgccaat | 2b | 30 sec | 63.1 |
| cemA^†^  [CDS](http://www.ncbi.nlm.nih.gov/entrez/viewer.fcgi?val=11990232&from=60553&to=61245&view=gbwithparts) | 5’ gaataccaggcaatccgaaa  3’ catgtgtcttcggtttctcttt | 2a | 30 sec | 52.4 |
| clpP*^†^ [CDS](http://www.ncbi.nlm.nih.gov/entrez/viewer.fcgi?val=11990232&from=69554&to=70204&view=gbwithparts) | 5’tcgttgcgagatcacaaatc  3’ tgtcaccgtttgcatcgtat | 2a | 30 sec | 59.5 |
| infA^†^  [CDS](http://www.ncbi.nlm.nih.gov/entrez/viewer.fcgi?val=11990232&from=78085&to=78408&view=gbwithparts) | 5’cccgagagaagcaaaagtca  3’ ggtgtcattctctaggcgaac | 2a | 30 sec | 49.9 |
| matK*^†^  [CDS](http://www.ncbi.nlm.nih.gov/entrez/viewer.fcgi?val=11990232&from=1674&to=3308&view=gbwithparts) | 5’tgcttcgattttctggggta  3’ ttgcaaggactgtggtatcg | 1 | 2 min to 30 sec | 54.6 |
| ndhA^†^ [CDS](http://www.ncbi.nlm.nih.gov/entrez/viewer.fcgi?val=11990232&itemID=90&view=gbwithparts) | 5’tggtcttctcatggcaggat  3’ ttgctagtacacaaaaagttaatggt | 2a | 30 sec | 59.0 |
| ndhB^†^  [CDS](http://www.ncbi.nlm.nih.gov/entrez/viewer.fcgi?val=11990232&itemID=100&view=gbwithparts) | 5’atcgggacttttcggagatt  3’ ccatggaagagaagcaaatga | 3 |  |  |
| ndhC^†^ [CDS](http://www.ncbi.nlm.nih.gov/entrez/viewer.fcgi?val=11990232&from=51855&to=52217&view=gbwithparts) | 5’ggacttttagccccggttag  3’ gtattcggaattgtaaccaagc | 2a | 30 sec | 52.5 |
| ndhD^†^ [CDS](http://www.ncbi.nlm.nih.gov/entrez/viewer.fcgi?val=11990232&from=110138&to=111640&view=gbwithparts) | 5’ggaattgttaccgcatgctc  3’ cccatgtgagatacggagga | 2b | 30 sec | 52.5 |
| ndhE^†^ [CDS](http://www.ncbi.nlm.nih.gov/entrez/viewer.fcgi?val=11990232&from=112473&to=112778&view=gbwithparts) | 5’gatcacaagccgaaacatgg  3’ aaaaattgcgaaaatgtctcc | 2a | 30 sec | 56.4 |
| ndhF^†^ [CDS](http://www.ncbi.nlm.nih.gov/entrez/viewer.fcgi?val=11990232&from=105072&to=107288&view=gbwithparts) | 5’tggagtttcgggatttgttc  3’ cgtgaagaggaaattgtgcag | 2a | 30 sec | 52.8 |
| ndhG^†^  [CDS](http://www.ncbi.nlm.nih.gov/entrez/viewer.fcgi?val=11990232&from=112993&to=113523&view=gbwithparts) | 5’atttgggggatttggtcttc  3’ cgaaaaggcagaataaattgg | 2a | 30 sec | 56.4 |
| ndhH^†^ [CDS](http://www.ncbi.nlm.nih.gov/entrez/viewer.fcgi?val=11990232&from=116456&to=117637&view=gbwithparts) | 5’aaaaaccttcgcccaatttt  3’ gacgaattttccatctccag | 2a | 30 sec | 52.8 |
| ndhI^†^  [CDS](http://www.ncbi.nlm.nih.gov/entrez/viewer.fcgi?val=11990232&from=113707&to=114249&view=gbwithparts) | 5’caattacatcggagcgtttc  3’ gcatacgcgaacacatacttc | 2a | 30 sec | 56.4 |
| ndhJ^†^  [CDS](http://www.ncbi.nlm.nih.gov/entrez/viewer.fcgi?val=11990232&from=50535&to=51014&view=gbwithparts) | 5’cgttttctgggtttggagaa  3’ gccagcctatccaactttca | 2a | 30 sec | 54.3 |
| ndhK^†^ [CDS](http://www.ncbi.nlm.nih.gov/entrez/viewer.fcgi?val=11990232&from=51118&to=51864&view=gbwithparts) | 5’gcgcgagaaataattgagga  3’ gagtactgcgccgaacataa | 2a | 30 sec | 54.3 |
| petA^†^ [CDS](http://www.ncbi.nlm.nih.gov/entrez/viewer.fcgi?val=11990232&from=61485&to=62447&view=gbwithparts) | 5’agtgcttcaggatccattgc  3’ tcgtacaattgaaccttttcaaac | 2a | 30 sec | 58.2 |
| petB^†^ [CDS](http://www.ncbi.nlm.nih.gov/entrez/viewer.fcgi?val=11990232&itemID=50&view=gbwithparts) | 5’taatgacggaggccaacttt  3’ cgcctgtgacccaagttaat | 2a | 30 sec | 59.5 |
| petD^†^ [CDS](http://www.ncbi.nlm.nih.gov/entrez/viewer.fcgi?val=11990232&itemID=51&view=gbwithparts) | 5’gggagttaacaaagaaacctgact  3’ aattatgtcccatccctttagc | 2a | 30 sec | 56.4 |
| petE^†^ [CDS](http://www.ncbi.nlm.nih.gov/entrez/viewer.fcgi?val=11990232&from=65611&to=65724&view=gbwithparts) | 5’tttctatttggaatcgtcttaggc  3’ actgatccccacgcctgtat | 2a | 30 sec | 59.3 |
| petN^†^ [CDS](http://www.ncbi.nlm.nih.gov/entrez/viewer.fcgi?val=11994090&from=19081&to=19170&view=gbwithparts) | 5’ aagtctcacttgggctgctt  3’ agtccactcctcccccatac | 2a | 30 sec | 56.4 |
| psaA^†^ [CDS](http://www.ncbi.nlm.nih.gov/entrez/viewer.fcgi?val=11990232&from=41352&to=43604&view=gbwithparts) | 5’ggaaaatgcagtcggatgtt  3’ agaaatctcgaagccaacca | 2b | 30 sec | 56 |
| psaB^†^ [CDS](http://www.ncbi.nlm.nih.gov/entrez/viewer.fcgi?val=11990232&from=39119&to=41326&view=gbwithparts) | 5’ggaccccactactcgtcgta  3’ atccggacgtccatagaaaga | 2a | 30 sec | 58.2 |
| psaC^†^ [CDS](http://www.ncbi.nlm.nih.gov/entrez/viewer.fcgi?val=11990232&from=111760&to=112005&view=gbwithparts) | 5’tacgagcttgcccaacagat  3’ cccacacaatcttcggttct | 2a | 30 sec | 48.1 |
| psaI^†^ [CDS](http://www.ncbi.nlm.nih.gov/entrez/viewer.fcgi?val=11990232&from=59193&to=59303&view=gbwithparts) | 5’cttaccctctattttcgtacctttag  3’ tgcacataaagaaataaggaagtca | 2a | 30 sec | 49.9 |
| psaJ^†^ [CDS](http://www.ncbi.nlm.nih.gov/entrez/viewer.fcgi?val=11990232&from=66513&to=66641&view=gbwithparts) | 5’cacccgtgctaagtactctatgg  3’ gggaatgacaaagcatctgg | 1 | 2 min to 30 sec | 52.4 |
| psbA^†^ [CDS](http://www.ncbi.nlm.nih.gov/entrez/viewer.fcgi?val=11990232&from=89&to=1150&view=gbwithparts) | 5’gtggctgctcacggttattt  3’ ccaagcagccaagaagaagt | 1 | 2 min to 30 sec | 52.5 |
| psbB^†^ [CDS](http://www.ncbi.nlm.nih.gov/entrez/viewer.fcgi?val=11990232&from=70706&to=72232&view=gbwithparts) | 5’aaggacgcgagctttttgta  3’ ccaccgttacgcctacttgt | 1 | 2 min to 30 sec | 55.1 |
| psbC^†^ [CDS](http://www.ncbi.nlm.nih.gov/entrez/viewer.fcgi?val=11990232&from=10092&to=11513&view=gbwithparts) | 5’ccgacgggtttaggtaaatatc  3’ gaaggtcccaaaaacgcata | 1 | 2 min to 30 sec | 52.5 |
| psbD^†^ [CDS](http://www.ncbi.nlm.nih.gov/entrez/viewer.fcgi?val=11990232&from=9083&to=10144&view=gbwithparts) | 5’ttccgtgcttttaacccaac  3’ ggaaaaagcaacaccaaaga | 1 | 2 min to 30 sec | 52.5 |
| psbE^†^ [CDS](http://www.ncbi.nlm.nih.gov/entrez/viewer.fcgi?val=11990232&from=63864&to=64115&view=gbwithparts) | 5’ctattcattgcgggttggtt  3’ gaattccttgtcggctttcc | 2b | 30 sec | 52.4 |
| psbF^†^ [CDS](http://www.ncbi.nlm.nih.gov/entrez/viewer.fcgi?val=11990232&from=63734&to=63853&view=gbwithparts) | 5’tgaccatagatcgaacctatcc  3’ tgaactgcattgctgatattg | 1 | 2 min to 30 sec | 53 |
| psbH^†^ [CDS](http://www.ncbi.nlm.nih.gov/entrez/viewer.fcgi?val=11990232&from=72790&to=73011&view=gbwithparts) | 5’tcggaatatgggaaagttgc  3’ tatcgcgaataaagccattg | 1 | 2 min to 30 sec | 52.5 |
| psbI^†^ [CDS](http://www.ncbi.nlm.nih.gov/entrez/viewer.fcgi?val=11990232&from=7775&to=7885&view=gbwithparts) | 5’gtgatattctttgtttccctcttt  3’ tactcctcacgcccaggat | 1 | 2 min to 30 sec | 54.3 |
| psbJ^†^  [CDS](http://www.ncbi.nlm.nih.gov/entrez/viewer.fcgi?val=11990232&from=63347&to=63469&view=gbwithparts) | 5’ggtactgtaactggtattcttgtgat  3’ agagatgaacccaatccaga | 2a | 30 sec | 52.5 |
| psbK^†^ [CDS](http://www.ncbi.nlm.nih.gov/entrez/viewer.fcgi?val=11990232&from=7199&to=7384&view=gbwithparts) | 5’ttttcttcgccaaattaccc  3’ tgccaaacaaacgctaatagaa | 1 | 2 min to 30 sec | 52.5 |
| psbL^†^  [CDS](http://www.ncbi.nlm.nih.gov/entrez/viewer.fcgi?val=11990232&from=63595&to=63711&view=gbwithparts) | 5’gacacaatcaaacccgaat  3’ ttggaaaataaaacagcaagtacaa | 2b | 30 sec | 52.5 |
| psbM^†^ [CDS](http://www.ncbi.nlm.nih.gov/entrez/viewer.fcgi?val=11990232&from=18178&to=18282&view=gbwithparts) | 5’cagcattgttcattctagttcct  3’ aatcattttgactggctgttttt | 2a | 30 sec | 60.9 |
| psbN^†^ [CDS](http://www.ncbi.nlm.nih.gov/entrez/viewer.fcgi?val=11990232&from=72555&to=72686&view=gbwithparts) | 5’cagcaactttagtcgccatctc  3’ gcccaaacgcggtatataag | 2a | 30 sec | 52.4 |
| rbcL^†^  [CDS](http://www.ncbi.nlm.nih.gov/entrez/viewer.fcgi?val=11990232&from=56874&to=58304&view=gbwithparts) | 5’ctacgcggtggacttgattt  3’ atttcaccagtttcggcttg | 1 | 2 min to 30 sec | 53.9 |
| rpl2^†^  [CDS](http://www.ncbi.nlm.nih.gov/entrez/viewer.fcgi?val=11990232&itemID=63&view=gbwithparts) | 5’aacacctatcccgagcacac  3’ aaggtcgtaatgccagagga | 3 |  |  |
| rpl14^†^  [CDS](http://www.ncbi.nlm.nih.gov/entrez/viewer.fcgi?val=11990232&from=79038&to=79409&view=gbwithparts) | 5’cataggagccgctggtaatc  3’ tcgtcgcctttgaattcttt | 2a | 30 sec | 56.1 |
| rpl16^†^  [CDS](http://www.ncbi.nlm.nih.gov/entrez/viewer.fcgi?val=11990232&itemID=58&view=gbwithparts) | 5’ccatcgactataaccccaaaa  3’ catatttttccaccacgacg | 2a | 30 sec | 54.7 |
| rpl20^†^  [CDS](http://www.ncbi.nlm.nih.gov/entrez/viewer.fcgi?val=11990232&from=68268&to=68627&view=gbwithparts) | 5’gcgttcatttgcctcaaact  3’ tgatccacaaacgacgaaaa | 1 | 2 min to 30 sec | 55.3 |
| rpl23^†^ [CDS](http://www.ncbi.nlm.nih.gov/entrez/viewer.fcgi?val=11990232&from=68268&to=68627&view=gbwithparts) | 5’tgggtcgaactcttctttgg  3’ ccggttgaagggtaatgatc | 2a | 30 sec | 54.3 |
| rpl32^†^  [CDS](http://www.ncbi.nlm.nih.gov/entrez/viewer.fcgi?val=11990232&from=108127&to=108306&view=gbwithparts) | 5’aagcgtattcgtaaaaatctttgg  3’ ctcgttgcccctagaaaatg | 2a | 30 sec | 52.9 |
| rpl33^†^ [CDS](http://www.ncbi.nlm.nih.gov/entrez/viewer.fcgi?val=11990232&from=66998&to=67198&view=gbwithparts) | 5’ aaagaatcgccacaatacgc  3’ tgagtggtatgcttgcgaca | 2a | 30 sec | 56 |
| rpl36^†^ [CDS](http://www.ncbi.nlm.nih.gov/entrez/viewer.fcgi?val=11990232&from=77871&to=77984&view=gbwithparts) | 5’ tgaaaataagagcttccgttcg  3’ tgtttatgcttcggattgga | 2b | 30 sec | 56 |
| rpoA^†^  [CDS](http://www.ncbi.nlm.nih.gov/entrez/viewer.fcgi?val=11990232&from=76170&to=77189&view=gbwithparts) | 5’ccaccccttttaacctttca  3’ ttggcccttttgagacaatta | 1 | 2 min to 30 sec | 52.4 |
| rpoB*^†^ [CDS](http://www.ncbi.nlm.nih.gov/entrez/viewer.fcgi?val=11990232&from=21468&to=24695&view=gbwithparts) | 5’cgaaaccgactccacaaact  3’ ccatgaaccgtttgtgtcaa | 1 | 2 min to 30 sec | 52.7 |
| rpoC1*^†^ [CDS](http://www.ncbi.nlm.nih.gov/entrez/viewer.fcgi?val=11990232&from=24733&to=26784&view=gbwithparts) | 5’ggtaaagaggggaggtttcg  3’ ttgatgtaatgaaagcgaagga | 1 | 2 min to 30 sec | 50.9 |
| rpoC2*^†^ [CDS](http://www.ncbi.nlm.nih.gov/entrez/viewer.fcgi?val=11990232&from=26983&to=31566&view=gbwithparts) | 5’ttggtaccggattccaaaaa  3’ catttccgacgcgaatagat | 2a | 30 sec | 64.7 |
| rps2^†^  [CDS](http://www.ncbi.nlm.nih.gov/entrez/viewer.fcgi?val=11990232&from=31858&to=32568&view=gbwithparts) | 5’gcagcaagtcagggaaaaa  3’ ctttttgggagatggtggaa | 2a | 30 sec | 54.7 |
| rps3^†^  [CDS](http://www.ncbi.nlm.nih.gov/entrez/viewer.fcgi?val=11990232&from=81113&to=81787&view=gbwithparts) | 5’gggtcgtctagcaggaaaag  3’ cgaattgtttggaggggtag | 1 | 2 min to 30 sec | 50.7 |
| rps8^†^  [CDS](http://www.ncbi.nlm.nih.gov/entrez/viewer.fcgi?val=11990232&from=78488&to=78898&view=gbwithparts) | 5’gacctggcttacgaatttatgc  3’ cccccaattctgtttagtcg | 3 | 30 sec | 52.9 |
| rps11^†^  [CDS](http://www.ncbi.nlm.nih.gov/entrez/viewer.fcgi?val=11990232&from=77252&to=77683&view=gbwithparts) | 5’tacagtgggtttgcaacgag  3’ gcggcataggtgttacatcg | 2a | 30 sec | 56.5 |
| rps14^†^  [CDS](http://www.ncbi.nlm.nih.gov/entrez/viewer.fcgi?val=11990232&from=38663&to=38974&view=gbwithparts) | 5’ctagaacaacgtgggccatt  3’ tatcgggtttcagagctatcaat | 3 |  |  |
| rps15^†^  [CDS](http://www.ncbi.nlm.nih.gov/entrez/viewer.fcgi?val=11990232&from=104729&to=104965&view=gbwithparts) | 5’ cggagacttgcttcacattt  3’ cgtttgctggcttatttgg | 3 |  |  |
| rps16^†^  [CDS](http://www.ncbi.nlm.nih.gov/entrez/viewer.fcgi?val=11990232&itemID=3&view=gbwithparts) | 5’ctgccgaggcaatcatattt  3’ cgtcgtggtggaaaaatatg | 2a | 30 sec | 55.3 |
| rps18^†^  [CDS](http://www.ncbi.nlm.nih.gov/entrez/viewer.fcgi?val=11990232&from=67532&to=68044&view=gbwithparts) | 5’ aggcaatttcaataattactggtc  3’ cacaaaagttcaattccaatcg | 2a | 30 sec | 46.0 |
| NAPD-ME GenBank: [J05130](http://www.ncbi.nlm.nih.gov/entrez/viewer.fcgi?val=J05130) | 5’ ctcccgtctaccgcaactac  3’ cctctccagcagcacca | 2a | 30 sec | 52.9 |
| NAPD-MDH GenBank: [AY105634](http://www.ncbi.nlm.nih.gov/entrez/viewer.fcgi?db=nuccore&id=21208712) | 5’ tcacctgctgttcaaactcg  3’ ggatacagcgagtcctccag | 2b | 30 sec | 61.9 |
| 4.5S GenBank: [NC_001666](http://www.ncbi.nlm.nih.gov/entrez/viewer.fcgi?val=NC_001666) [rRNA](http://www.ncbi.nlm.nih.gov/entrez/viewer.fcgi?val=11994090&from=102041&to=102135&view=gbwithparts)  (optimal primer set) | 5’ ggtgtcaagtggaagtgcag  3’ caaatcgttcgttcgttagg | 3 | 30 sec | 52.7 |
| 16S GenBank: [NC_001666](http://www.ncbi.nlm.nih.gov/entrez/viewer.fcgi?val=NC_001666) [rRNA](http://www.ncbi.nlm.nih.gov/entrez/viewer.fcgi?val=11994090&from=126086&to=127576&view=gbwithparts) | 5’ cgcaaccctcgtgtttagtt  3’ cccattgtagcacgtgtgtc | 3 | 30 sec | 46.5 |
| 23S GenBank : [NC_001666](http://www.ncbi.nlm.nih.gov/entrez/viewer.fcgi?val=NC_001666) [rRNA](http://www.ncbi.nlm.nih.gov/entrez/viewer.fcgi?val=11994090&from=99062&to=101945&view=gbwithparts) | 5’ cggggagttgaaaataagca  3’ gatgtttcagttcgccaggt | 3 | 30 sec | 52.7 |

* These primer sequences have appeared in a previously published report [24] by two of the authors. Their optimization conditions

however are unique to this report.

^†^ GenBank: [X86563](http://www.ncbi.nlm.nih.gov/entrez/viewer.fcgi?db=nucleotide&val=X86563)
