# Supplementary material for: A plastome primer set for comprehensive quantitative real time RT-PCR analysis of Zea mays: a starter primer set for other Poaceae species
Source: Plant Methods. 2008 Jun 2;4:14. doi: 10.1186/1746-4811-4-14 (PMC2453112; doi:10.1186/1746-4811-4-14)
Supplement: Additional File 2 — in silico comparison of maize primers to other plastome sequences. Primer sequences were compared to representative plastomes from the BEP and PACCAD clades, as well as Arabidopsis. Success was gauged by overall percent homology and the presence of mismatched bases at the 3' end of the primers. These data are summarized in Figure 3. [file 1746-4811-4-14-S2.doc]

| Common Name | Maize | Sugarcane | Sorghum | Lolium (perrenial ryegrass) | Barley | Wheat | Arabidopsis (thale cress) |
| --- | --- | --- | --- | --- | --- | --- | --- |
| Taxonomy | Liliopsida; commelinids; Poales; Poaceae; PACCAD clade; Panicoideae; Andropogoneae; Zea mays | Liliopsida; commelinids; Poales; Poaceae; PACCAD clade; Panicoideae; Andropogoneae; Saccharum officinarum | Liliopsida; commelinids; Poales; Poaceae; PACCAD clade; Panicoideae; Andropogoneae; Sorghum | *Liliopsida; commelinids; Poales; Poaceae; BEP clade; Pooideae; Poeae; Lolium perenne* | *Liliopsida; commelinids; Poales; Poaceae; BEP clade; Pooideae; Triticeae; Hordeum vulgare* | *Liliopsida; commelinids; Poales; Poaceae; BEP clade; Pooideae; Triticeae; Triticum aestivum* | *Magnoliophyta….Arabidopsis thaliana* |
| gene name |  |  |  |  |  |  |  |
| atpA  ^†^ CDS | 5’ acccatcgcccattaataca | 5' match | 5' match | 5' match | 5' match | 5' match | no match |
|  | 3’ aatttgcagaagggacgaga | 3' match | 3' match | no match | no match | no match |  |
| [atpB† CDS](http://www.ncbi.nlm.nih.gov/entrez/viewer.fcgi?val=11990232&from=54618&to=56114&view=gbwithparts) | 5’ gggttgatgagaggaatgga | 5' match | 5' match | 5' match | 5' match | 5' match | no match |
|  | 3’ cgttaaaaattcgtccgaga | 3' match | 3' match | 3' 19 of 20 match | 3' 19 of 20 match | no match |  |
| [atpE† CDS](http://www.ncbi.nlm.nih.gov/entrez/viewer.fcgi?val=11990232&from=54208&to=54621&view=gbwithparts) | 5’ cggttctgtggagtggtttt | 5' match | 5' match | 5' 19 of 20 match | 5' 19 of 20 match | 5' 19 of 20 match | no match |
|  | 3’ gcctgttgagcttcttctgg | 3' match | 3' match | 3' last 16 of 17 match | 3' last 16 of 17 match | no match |  |
| [atpF† CDS](http://www.ncbi.nlm.nih.gov/entrez/viewer.fcgi?val=11990232&itemID=18&view=gbwithparts) | 5’ctgggagtttcgggcttaat | 5' match | 5' match | 5' 19 of 20 match | 5' 19 of 20 match | 5' 19 of 20 match | 5' match (19 of 20) |
|  | 3’ aactcgcacacactcccttt | 3' match | 3' match | 3' match | 3' match | 3' match | 3' no match |
| [atpH† CDS](http://www.ncbi.nlm.nih.gov/entrez/viewer.fcgi?val=11990232&from=34381&to=34626&view=gbwithparts) | 5’cagaagcagaaggtaaaataagagg | 5' match | 5' match | 5' 24 of 25 match | 5' 24 of 25 match | 5' 24 of 25 match | no match |
|  | 3’ tgccacaaccagtccataaa | 3' match | 3' match | no match | no match | no match |  |
| [atpI† CDS](http://www.ncbi.nlm.nih.gov/entrez/viewer.fcgi?val=11990232&from=32820&to=33563&view=gbwithparts) | 5’ccaaccccaatccttttacc | 5' match | 5' match | 5' 19 of 20 match | 5' match | 5' 19 of 20 match | 5' match (19 of 20) |
|  | 3’ cgactaattcatccgccaat | 3' match | 3' match | 3' match | 3' match | 3' match | no match |
| [cemA† CDS](http://www.ncbi.nlm.nih.gov/entrez/viewer.fcgi?val=11990232&from=60553&to=61245&view=gbwithparts) | 5’ gaataccaggcaatccgaaa | 5' match | 5' match | 5' 19 of 20 match | 5' match | 5 match | no match |
|  | 3’ catgtgtcttcggtttctcttt | 3' match | 3' match | no match | no match | no match |  |
| [clpP*† CDS](http://www.ncbi.nlm.nih.gov/entrez/viewer.fcgi?val=11990232&from=69554&to=70204&view=gbwithparts) | 5’tcgttgcgagatcacaaatc | 5' 19 of 20 match | 5' 19 of 20 match | 5' 19 of 20 match | 5' 19 of 20 match | 5' 19 of 20 match | no hits at all |
|  | 3’ tgtcaccgtttgcatcgtat | 3' match | 3' match | 3' match | 3' match | 3' match |  |
| [infA† CDS](http://www.ncbi.nlm.nih.gov/entrez/viewer.fcgi?val=11990232&from=78085&to=78408&view=gbwithparts) | 5’cccgagagaagcaaaagtca | 5' match | 5' 18 match | 5' match | 5' 1st 18 match | 5' 1st 18 match | no match |
|  | 3’ ggtgtcattctctaggcgaac | 3' match | 3' 19 match | 3' 16 of 17 match | 3' 19 of 20 match | 3' 19 of 20 match |  |
| [matK*† CDS](http://www.ncbi.nlm.nih.gov/entrez/viewer.fcgi?val=11990232&from=1674&to=3308&view=gbwithparts) | 5’tgcttcgattttctggggta | 5' match | 5' match | no match | 5' 1st 16 of 17 match | 5' 19 of 20 match | no match |
|  | 3’ ttgcaaggactgtggtatcg | 3' last 18 of 20 match (3' to 5') | 3' 19 of 20 match |  | no match | no match |  |
| [ndhA† CDS](http://www.ncbi.nlm.nih.gov/entrez/viewer.fcgi?val=11990232&itemID=90&view=gbwithparts) | 5’tggtcttctcatggcaggat | 5' match | 5' match | no match | no match | no match | no hits at all |
|  | 3’ ttgctagtacacaaaaagttaatggt | 3' match | 3' match | 3' 24 of 25 match | 3' 24 of 25 | 3' 24 of 25 |  |
| [ndhB† CDS](http://www.ncbi.nlm.nih.gov/entrez/viewer.fcgi?val=11990232&itemID=100&view=gbwithparts) | 5’atcgggacttttcggagatt | 5' match | 5' match | 5' match | 5' match | no match | no match |
|  | 3’ ccatggaagagaagcaaatga | 3' 20 of 21 match | 3' 20 of 21 match | 3' 20 of 21 match | 3' 20 of 21 match | 3' 20 of 21 | 3' 19 of 20 match |
| [ndhC† CDS](http://www.ncbi.nlm.nih.gov/entrez/viewer.fcgi?val=11990232&from=51855&to=52217&view=gbwithparts) | 5’ggacttttagccccggttag | 5' match | 5' match | 5' last 16 of 20 match | 5' last 16 of 20 match | 5' match | no match |
|  | 3’ gtattcggaattgtaaccaagc | 3' match | 3' match | 3' 20 of 21 match | 3' 20 of 21 match | 3' 20 of 21 match | 3' 1st 14 (5' to 3') |
| [ndhD† CDS](http://www.ncbi.nlm.nih.gov/entrez/viewer.fcgi?val=11990232&from=110138&to=111640&view=gbwithparts) | 5’ggaattgttaccgcatgctc | 5' match | 5' match | 5' 19 of 20 match | 5' 19 of 20 match | 5' 19 of 20 match | no hits at all |
|  | 3’ cccatgtgagatacggagga | 3' match | 3' match | no match | 3' 19 of 20 match | 3' 19 of 20 match |  |
| [ndhE† CDS](http://www.ncbi.nlm.nih.gov/entrez/viewer.fcgi?val=11990232&from=112473&to=112778&view=gbwithparts) | 5’gatcacaagccgaaacatgg | 5' match | 5' match | 5' match | 5' match | 5' match | no match |
|  | 3’ aaaaattgcgaaaatgtctcc | 3' match | 3' match | 3' match | no match | 3' 20 of 21 match |  |
| [ndhF† CDS](http://www.ncbi.nlm.nih.gov/entrez/viewer.fcgi?val=11990232&from=105072&to=107288&view=gbwithparts) | 5’tggagtttcgggatttgttc | 5' match | 5' match | 5' last 16 of 17 (5' 3') | 5' last 16 of 17 (5' 3') | no match | no match |
|  | 3’ cgtgaagaggaaattgtgcag | 3' match | 3' match | 3' 10 of last 11, 1 from 3' | 3' 10 of last 11, 1 from 3' |  |  |
| [ndhG† CDS](http://www.ncbi.nlm.nih.gov/entrez/viewer.fcgi?val=11990232&from=112993&to=113523&view=gbwithparts) | 5’atttgggggatttggtcttc | 5' 19 of 20 match | 5' 19 0f 20 match | no match | no match | 5' 19 of 20 match | no match |
|  | 3’ cgaaaaggcagaataaattgg | 3' match | 3' match | 3' 1st 18 of 20 (5' to 3') | 3' 1st 18 of 20 (5' to 3') | no match |  |
| [ndhH† CDS](http://www.ncbi.nlm.nih.gov/entrez/viewer.fcgi?val=11990232&from=116456&to=117637&view=gbwithparts) | 5’aaaaaccttcgcccaatttt | 5' match | 5' 19 of 20 match | 5' 19 of 20 match | 5' 19 of 20 match | 5' match | 5' 1st 13 (5' to 3') |
|  | 3’ gacgaattttccatctccag | 3' match | 3' match | 3' match | 3' 19 of 20 match | 3' 19 of 20 match | no match |
| [ndhI† CDS](http://www.ncbi.nlm.nih.gov/entrez/viewer.fcgi?val=11990232&from=113707&to=114249&view=gbwithparts) | 5’caattacatcggagcgtttc | 5' match | 5' match | 5' no hits at all | 5' no hits at all | 5' match | no match |
|  | 3’ gcatacgcgaacacatacttc | 3' match | 3' match | 3' 19 of 20 match | 3' match | 3' no hits at all | 3' 19 of 20 match |
| [ndhJ† CDS](http://www.ncbi.nlm.nih.gov/entrez/viewer.fcgi?val=11990232&from=50535&to=51014&view=gbwithparts) | 5’cgttttctgggtttggagaa | 5' match | no match | 5' no hits at all | 5' no hits at all | 5' 1st 10 match | 5' 1st 17 (5' to 3') |
|  | 3’ gccagcctatccaactttca | 3' match | 3' match | 3' 19 of 20 match | 3' match | 3' match | no match |
| [ndhK† CDS](http://www.ncbi.nlm.nih.gov/entrez/viewer.fcgi?val=11990232&from=51118&to=51864&view=gbwithparts) | 5’gcgcgagaaataattgagga | 5' match | 5' match | 5' 19 of last 20 (5' to 3') | 5' 19 of last 20 (5' to 3') | 5' 18 of 19 (3' to 5') | 5' 1st 11 (5' to 3') |
|  | 3’ gagtactgcgccgaacataa | 3' match | 3' match | 3' 19 of 20 match | 3' 19 of 20 match | 3' 19 of 20 match | no match |
| [petA† CDS](http://www.ncbi.nlm.nih.gov/entrez/viewer.fcgi?val=11990232&from=61485&to=62447&view=gbwithparts) | 5’agtgcttcaggatccattgc | 5' match | 5' match | 5' 1st 18 match | 5' 18 of 1st 19(5' to 3') | 5' 2 thru 18 match | no match |
|  | 3’ tcgtacaattgaaccttttcaaac | 3' match | 3' match | 3' 24 of 25 match | 3' match | 3' match | 3' 18 of 1st 19 match |
| [petB† CDS](http://www.ncbi.nlm.nih.gov/entrez/viewer.fcgi?val=11990232&itemID=50&view=gbwithparts) | 5’taatgacggaggccaacttt | 5' match | 5' match | 5' 19 of 20 match | 5' 19 of 20 match | 5' 19 of 20 match | no match |
|  | 3’ cgcctgtgacccaagttaat | 3' match | 3' match | 3' 1st 17 of 18 (3' to 5') | 3' 1st 17 of 18 (3' to 5') | 3' 1st 17 match | 3' match of first 11 5' to 3' |
| [petD† CDS](http://www.ncbi.nlm.nih.gov/entrez/viewer.fcgi?val=11990232&itemID=51&view=gbwithparts) | 5’gggagttaacaaagaaacctgact | no match | no match | no match | no match | no match | no match |
|  | 3’ aattatgtcccatccctttagc | 3' match | 3' match | 3' match | 3' match | 3' match | 3' 1st 12 (5' to 3') |
| [petE† CDS](http://www.ncbi.nlm.nih.gov/entrez/viewer.fcgi?val=11990232&from=65611&to=65724&view=gbwithparts) | 5’tttctatttggaatcgtcttaggc | 5' match | 5' match | 5' match | 5' match | 5' match | no match |
|  | 3’ actgatccccacgcctgtat | 3' match | 3' match | 3' match | 3' match | 3' match |  |
| [petN† CDS](http://www.ncbi.nlm.nih.gov/entrez/viewer.fcgi?val=11994090&from=19081&to=19170&view=gbwithparts) | 5’ aagtctcacttgggctgctt | 5' 19 of 20 match | 5' 19 of 20 match | 5' 19 of 20 match | 5' 19 of 20 match | 5' 19 of 20 match | no match |
|  | 3’ agtccactcctcccccatac | 3' match | 3' match | 3' match | 3' 19 of 20 match | 3' 19 of 20 match |  |
| [psaA† CDS](http://www.ncbi.nlm.nih.gov/entrez/viewer.fcgi?val=11990232&from=41352&to=43604&view=gbwithparts) | 5’ggaaaatgcagtcggatgtt | 5' match | 5' match | 5' match | 5' match | 5' match | 5' 18 of 19 (5' to 3') |
|  | 3’ agaaatctcgaagccaacca | 3' match | 3' match | no match | no match | no match | no match |
| [psaB† CDS](http://www.ncbi.nlm.nih.gov/entrez/viewer.fcgi?val=11990232&from=39119&to=41326&view=gbwithparts) | 5’ggaccccactactcgtcgta | 5' match | 5' match | 5' match | 5' match | 5' match | 5' match |
|  | 3’ atccggacgtccatagaaaga | 3' match | 3' match | 3' match | 3' match | 3' match | 3' match of first 11 5' to 3' |
| [psaC† CDS](http://www.ncbi.nlm.nih.gov/entrez/viewer.fcgi?val=11990232&from=111760&to=112005&view=gbwithparts) | 5’tacgagcttgcccaacagat | 5' match | 5' match | no match | no match | no match | no match |
|  | 3’ cccacacaatcttcggttct | 3' match | 3' match | 3' 19 of 20 match | 3' match | 3' match |  |
| [psaI† CDS](http://www.ncbi.nlm.nih.gov/entrez/viewer.fcgi?val=11990232&from=59193&to=59303&view=gbwithparts) | 5’cttaccctctattttcgtacctttag | 5' match | 5' match | 5' 22 of 24 match | 5'no hits | 5' 22 of 24 match | 5' 22 of 24 match |
|  | 3’ tgcacataaagaaataaggaagtca | 3' match | 3' match | no match | 3' match | 3' 23 of 24 match | no match |
| [psaJ† CDS](http://www.ncbi.nlm.nih.gov/entrez/viewer.fcgi?val=11990232&from=66513&to=66641&view=gbwithparts) | 5’cacccgtgctaagtactctatgg | 5' match | 5' match | 5' match | 5' match | 5' match | no match |
|  | 3’ gggaatgacaaagcatctgg | 3' match | 3' match | 3' match | 3' match | 3' match |  |
| [psbA† CDS](http://www.ncbi.nlm.nih.gov/entrez/viewer.fcgi?val=11990232&from=89&to=1150&view=gbwithparts) | 5’gtggctgctcacggttattt | 5' match | 5' match | 5' 19 of 20 match | 5' 19 of 20 match | 5' 19 of 20 match | no match |
|  | 3’ ccaagcagccaagaagaagt | 3' match | 3' match | 3' match | 3' match | 3' match |  |
| [psbB† CDS](http://www.ncbi.nlm.nih.gov/entrez/viewer.fcgi?val=11990232&from=70706&to=72232&view=gbwithparts) | 5’aaggacgcgagctttttgta | 5' match | 5' match | 5' 19 of 20 match | 5' 19 of 20 match | 5' 19 of 20 match | no hits at all |
|  | 3’ ccaccgttacgcctacttgt | 3' match | 3' match | 3' match | 3' match | 3' match |  |
| [psbC† CDS](http://www.ncbi.nlm.nih.gov/entrez/viewer.fcgi?val=11990232&from=10092&to=11513&view=gbwithparts) | 5’ccgacgggtttaggtaaatatc | 5' match | 5' match | no match | no match | no match | no match |
|  | 3’ gaaggtcccaaaaacgcata | 3' 19 of 20 match | 3' 19 of 20 match | 3' match | 3' match | 3' match |  |
| [psbD† CDS](http://www.ncbi.nlm.nih.gov/entrez/viewer.fcgi?val=11990232&from=9083&to=10144&view=gbwithparts) | 5’ttccgtgcttttaacccaac | 5' match | 5' match | 5' 19 of 20 match | 5' match | 5' match | 5' match |
|  | 3’ ggaaaaagcaacaccaaaga | 3' match | 3' match | 3' match | 3' match | 3' match | 3' 19 of 20 match |
| [psbE† CDS](http://www.ncbi.nlm.nih.gov/entrez/viewer.fcgi?val=11990232&from=63864&to=64115&view=gbwithparts) | 5’ctattcattgcgggttggtt | 5' match | 5' match | 5' 19 of 20 match | 5' match | 5' 19 of 20 match | 5' 19 of 20 match |
|  | 3’ gaattccttgtcggctttcc | 3' match | 3' match | 3' 19 of 20 match | 3' match | 3' match | no match |
| [psbF† CDS](http://www.ncbi.nlm.nih.gov/entrez/viewer.fcgi?val=11990232&from=63734&to=63853&view=gbwithparts) | 5’tgaccatagatcgaacctatcc | 5' match | 5' match | 5' match | 5' match | 5' match | no match |
|  | 3’ tgaactgcattgctgatattg | 3' match | 3' match | 3' match | 3' match | 3' match | 3' match |
| [psbH† CDS](http://www.ncbi.nlm.nih.gov/entrez/viewer.fcgi?val=11990232&from=72790&to=73011&view=gbwithparts) | 5’tcggaatatgggaaagttgc | 5' match | 5' match | no match | 5' 1st 17 match | 5' 1st 17 match | no match |
|  | 3’ tatcgcgaataaagccattg | 3' match | 3' match | 3' 1st 15 match | 3' 1st 15 match (3' to 5') | 3' 1st 15 match |  |
| [psbI† CDS](http://www.ncbi.nlm.nih.gov/entrez/viewer.fcgi?val=11990232&from=7775&to=7885&view=gbwithparts) | 5’gtgatattctttgtttccctcttt | 5' match | 5' match | no match | 5' 23 of 24 match | 5' 23 of 24 match | no match |
|  | 3’ tactcctcacgcccaggat | 3' match | 3' match | 3' 19 of 20 match | 3' 19 of 21 match | no match |  |
| [psbJ† CDS](http://www.ncbi.nlm.nih.gov/entrez/viewer.fcgi?val=11990232&from=63347&to=63469&view=gbwithparts) | 5’ggtactgtaactggtattcttgtgat | 5' match | 5' match | 5' 25 of 26 match | no match | no match | 5' 25 of 26 match |
|  | 3’ agagatgaacccaatccaga | 3' match | 3' match | 3' match | 3' match | 3' match | no match |
| [psbK† CDS](http://www.ncbi.nlm.nih.gov/entrez/viewer.fcgi?val=11990232&from=7199&to=7384&view=gbwithparts) | 5’ttttcttcgccaaattaccc | 5' match | 5' match | no hits at all | 5' no hits at all | 5' 1st 16 match | no match |
|  | 3’ tgccaaacaaacgctaatagaa | 3' match | 3' match | 3' 20 of 21 match | 3' 21 of 22 match | 3' 20 of 21 match | 3' match 20 of 22 |
| [psbL† CDS](http://www.ncbi.nlm.nih.gov/entrez/viewer.fcgi?val=11990232&from=63595&to=63711&view=gbwithparts) | 5’gacacaatcaaacccgaat | 5' match | 5' match | 5' match | 5' match | 5' match | 5' match |
|  | 3’ ttggaaaataaaacagcaagtacaa | 3' match | 3' match | 3' match | 3' match | 3' 22 of 24 match | 3' match of 1st 22 (3' to 5') |
| [psbM† CDS](http://www.ncbi.nlm.nih.gov/entrez/viewer.fcgi?val=11990232&from=18178&to=18282&view=gbwithparts) | 5’cagcattgttcattctagttcct | 5' match | 5' match | no match | no match | no match | no match |
|  | 3’ aatcattttgactggctgttttt | 3' match | 3' match | 3' 20 of 22 match |  |  |  |
| [psbN† CDS](http://www.ncbi.nlm.nih.gov/entrez/viewer.fcgi?val=11990232&from=72555&to=72686&view=gbwithparts) | 5’cagcaactttagtcgccatctc | 5' match | 5' match | 5' match | 5' match | 5' match | 5' 19 of 21 match |
|  | 3’ gcccaaacgcggtatataag | 3' match | 3' match | 3' match | 3' match | 3' match | no match |
| [rbcL† CDS](http://www.ncbi.nlm.nih.gov/entrez/viewer.fcgi?val=11990232&from=56874&to=58304&view=gbwithparts) | 5’ctacgcggtggacttgattt | 5' match | 5' match | 5' 19 of 20 match | 5' 19 of 20 match | 5' 19 of 20 match | 5' 19 of 20 match |
|  | 3’ atttcaccagtttcggcttg | 3' 19 of 20 match | 3' 19 of 20 match | 3' 1st 10 match(3' to 5') | 3' 1st 11 match (3' to 5') | 3' 1st 10 match (3' to 5') | no match |
| [rpl2† CDS](http://www.ncbi.nlm.nih.gov/entrez/viewer.fcgi?val=11990232&itemID=63&view=gbwithparts) | 5’aacacctatcccgagcacac | 5' match | 5' match | 5' match | 5' match | 5' match | no match |
|  | 3’ aaggtcgtaatgccagagga | 3' match | 3' match | 3' 19 of 20 match | 3' 19 of 20 match | 3' 19 of 20 match | 3' match |
| [rpl14† CDS](http://www.ncbi.nlm.nih.gov/entrez/viewer.fcgi?val=11990232&from=79038&to=79409&view=gbwithparts) | 5’cataggagccgctggtaatc | 5' match | 5' match | 5' match | no match | no match | no match |
|  | 3’ tcgtcgcctttgaattcttt | 3' match | 3' match | 3' 10 of last 11, 1 from 3' | 3' 16 of 17 (3' to 5') |  |  |
| [rpl16† CDS](http://www.ncbi.nlm.nih.gov/entrez/viewer.fcgi?val=11990232&itemID=58&view=gbwithparts) | 5’ccatcgactataaccccaaaa | 5' match | 5' match | 5' 19 of 20 match | 5' match | 5' match | no match |
|  | 3’ catatttttccaccacgacg | 3' match | 3' match | 3' match | 3'match | 3' match |  |
| [rpl20† CDS](http://www.ncbi.nlm.nih.gov/entrez/viewer.fcgi?val=11990232&from=68268&to=68627&view=gbwithparts) | 5’gcgttcatttgcctcaaact | 5' match | 5' match | 5' match | 5' match | 5' match | no match |
|  | 3’ tgatccacaaacgacgaaaa | 3' match | 3' match | 3' match | 3'match | 3' match |  |
| [rpl23† CDS](http://www.ncbi.nlm.nih.gov/entrez/viewer.fcgi?val=11990232&from=68268&to=68627&view=gbwithparts) | 5’tgggtcgaactcttctttgg | 5' match | 5' match | 5' match | 5' match | 5' match | 5' match |
|  | 3’ ccggttgaagggtaatgatc | 3' match | 3' match | 3' match | 3' match | 3' match | 3' match 1st 11 (3' to 5') |
| [rpl32† CDS](http://www.ncbi.nlm.nih.gov/entrez/viewer.fcgi?val=11990232&from=108127&to=108306&view=gbwithparts) | 5’aagcgtattcgtaaaaatctttgg | 5' match | 5' match | 5' 23 of 24 match | 5' 23 of 24 match | 5' 22 of 23 match | 5' match 1st 17 (5' to 3') |
|  | 3’ ctcgttgcccctagaaaatg | no match | no match | 3' no match | 3' no match | no match | no match |
| [rpl33† CDS](http://www.ncbi.nlm.nih.gov/entrez/viewer.fcgi?val=11990232&from=66998&to=67198&view=gbwithparts) | 5’ aaagaatcgccacaatacgc | 5' match | 5' match | 5' 1st 18 match | 5' 1st 18 match | 5' 1st 18 match | no hits at all |
|  | 3’ tgagtggtatgcttgcgaca | 3' 19 of 20 match | 3' match | 3' 19 of 20 match | 3' 19 of 20 match | 3' 19 of 20 match |  |
| [rpl36† CDS](http://www.ncbi.nlm.nih.gov/entrez/viewer.fcgi?val=11990232&from=77871&to=77984&view=gbwithparts) | 5’ tgaaaataagagcttccgttcg | 5' match | 5' match | 5' match | 5' match | 5' match | 5' match 22 of 23 |
|  | 3’ tgtttatgcttcggattgga | 3' match | 3' match | 3' no match | 3' last 17 match (3' to 5') | 3' last 17 match (3' to 5') | no match |
| [rpoA† CDS](http://www.ncbi.nlm.nih.gov/entrez/viewer.fcgi?val=11990232&from=76170&to=77189&view=gbwithparts) | 5’ccaccccttttaacctttca | 5' match | 5' match | 5' 18 of 20 match | 5' 18 of 20 match | 5' 18 of 20 match | no match |
|  | 3’ ttggcccttttgagacaatta | 3' match | 3' match | 3' match | 3' 20 of 21 match | 3' 20 of 21 match | 3' 1st 12 (3' to 5')match |
| [rpoB*† CDS](http://www.ncbi.nlm.nih.gov/entrez/viewer.fcgi?val=11990232&from=21468&to=24695&view=gbwithparts) | 5’cgaaaccgactccacaaact | 5' match | 5' match | 5' 19 of 20 match | 5' 19 of 20 match | 5' 19 of 20 match | no hits at all |
|  | 3’ ccatgaaccgtttgtgtcaa | 3' match | 3' match | 3' 19 of 20 match | 3' 19 of 20 match | 3' 19 of 20 match |  |
| [rpoC1*† CDS](http://www.ncbi.nlm.nih.gov/entrez/viewer.fcgi?val=11990232&from=24733&to=26784&view=gbwithparts) | 5’ggtaaagaggggaggtttcg | 5' match | 5' match | 5' 1st 11 match | 5' 19 of 20 match | 5' 19 of 20 match | no match |
|  | 3’ ttgatgtaatgaaagcgaagga | 3' match | 3' match | 3' match | 3' match | 3' match |  |
| [rpoC2*† CDS](http://www.ncbi.nlm.nih.gov/entrez/viewer.fcgi?val=11990232&from=26983&to=31566&view=gbwithparts) | 5’ttggtaccggattccaaaaa | 5' match | 5' match | 5' match | 5' match | 5' match | no match |
|  | 3’ catttccgacgcgaatagat | 3' match | 3' match | 3' match | 3' match | 3' match |  |
| [rps2† CDS](http://www.ncbi.nlm.nih.gov/entrez/viewer.fcgi?val=11990232&from=31858&to=32568&view=gbwithparts) | 5’gcagcaagtcagggaaaaa | 5' match | 5' 19 of 20 match | 5' match | 5' match | 5' match | no match |
|  | 3’ ctttttgggagatggtggaa | 3' match | 3' match | 3' 19 of 20 match | 3' match | 3' match |  |
| [rps3† CDS](http://www.ncbi.nlm.nih.gov/entrez/viewer.fcgi?val=11990232&from=81113&to=81787&view=gbwithparts) | 5’gggtcgtctagcaggaaaag | 5' match | 5' match | no match | no match | 5' no match | no match |
|  | 3’ cgaattgtttggaggggtag | 3' match | 3' match |  |  | 3' last 17 match (3' to 5') |  |
| [rps8† CDS](http://www.ncbi.nlm.nih.gov/entrez/viewer.fcgi?val=11990232&from=78488&to=78898&view=gbwithparts) | 5’gacctggcttacgaatttatgc | 5' match | 5' match | 5' 19 of 20 match | 5' 19 of 20 match | 5' 19 of 20 match | no match |
|  | 3’ cccccaattctgtttagtcg | 3' match | 3' match | 3' match 1st 17 | 3' match | 3' match |  |
| [rps11† CDS](http://www.ncbi.nlm.nih.gov/entrez/viewer.fcgi?val=11990232&from=77252&to=77683&view=gbwithparts) | 5’tacagtgggtttgcaacgag | 5' match | 5' match | 5' 19 of 20 match | 5' 19 of 20 match | 5' 19 of 20 match | no hits at all |
|  | 3’ gcggcataggtgttacatcg | 3' match | 3' match | 3' last 19 of 20 match (3' to 5') | 3' match | 3' match |  |
| [rps14† CDS](http://www.ncbi.nlm.nih.gov/entrez/viewer.fcgi?val=11990232&from=38663&to=38974&view=gbwithparts) | 5’ctagaacaacgtgggccatt | no match | no match | no match | no match | no match | no hits at all |
|  | 3’ tatcgggtttcagagctatcaat |  |  |  |  |  |  |
| [rps15† CDS](http://www.ncbi.nlm.nih.gov/entrez/viewer.fcgi?val=11990232&from=104729&to=104965&view=gbwithparts) | 5’ cggagacttgcttcacattt | 5' match | 5' match | 5' match | 5' match | 5' match | no match |
|  | 3’ cgtttgctggcttatttgg | 3' match | 3' match | 3' match | 3' match | 3' match | 3' 1st 10 |
| [rps16† CDS](http://www.ncbi.nlm.nih.gov/entrez/viewer.fcgi?val=11990232&itemID=3&view=gbwithparts) | 5’ctgccgaggcaatcatattt | 5' match | 5' match | 5' 19 of 20 match | 5' 15 of 16 match | 5' 19 of 20 match | no match |
|  | 3’ cgtcgtggtggaaaaatatg | 3' match | 3' match | 3' match | 3' match | 3' match |  |
| [rps18† CDS](http://www.ncbi.nlm.nih.gov/entrez/viewer.fcgi?val=11990232&from=67532&to=68044&view=gbwithparts) | 5’ aggcaatttcaataattactggtc | 5' match | 5' match | no match | no match | no match | no match |
|  | 3’ cacaaaagttcaattccaatcg | 3' match | 3' match |  | 3' 19 of 20 match | 3' 1st 19 of 20 (3' to 5') | 3' 1st 13(3' to 5')match |
| [NAPD-ME GenBank: J05130](http://www.ncbi.nlm.nih.gov/entrez/viewer.fcgi?val=J05130) | 5’ ctcccgtctaccgcaactac | 5' no hit | 5' no hits | no match | no match | no match | no match |
|  | 3’ cctctccagcagcacca | 3' 1st 10 match | 3' 1st 10 (3' to 5') |  |  |  |  |
| [NAPD-MDH GenBank: AY105634](http://www.ncbi.nlm.nih.gov/entrez/viewer.fcgi?db=nuccore&id=21208712) | 5’ tcacctgctgttcaaactcg | ho hits | no hits | no hits at all | no hits | no hits found | no match |
|  | 3’ ggatacagcgagtcctccag | 5' match | 5' match | 5' match | 5' match | 5' match | 5' match |
| 4.5S GenBank: NC_001666 rRNA | 5’ ggtgtcaagtggaagtgcag | 3' match | 3' match | 3' match | 3' match | 3' match | No 3' match |
| (optimal primer set) | 3’ caaatcgttcgttcgttagg | 3' match | 3' match | 3' match | 3' match | 3' match | No 3' match |
| 16S GenBank: NC_001666 rRNA | 5’ cgcaaccctcgtgtttagtt | 5' match | 5' match | 5' match | 5' match | 5' match | 5' match |
|  | 3’ cccattgtagcacgtgtgtc | 3' match | 3' match | 3' match | 3'match | 3' match | 3' 19of 20 match |
| 23S GenBank : NC_001666 rRNA | 5’ cggggagttgaaaataagca | 5' match | 5' match | 5' match | 5' match | 5' match | 5' match |
|  | 3’ gatgtttcagttcgccaggt | 3' match | 3' match | 3' match | 3'match | 3' match | 3' match |
|  |  |  |  |  |  |  |  |
| The gene names need to line up consistently. Maybe left justification would be best for this column | | |  |  |  |  |  |
